# Losartan-induced Severe Hepatic Injury: A Case Report and Literature Review

**DOI:** 10.7759/cureus.4769

**Published:** 2019-05-28

**Authors:** Ravikaran Patti, Ankur Sinha, Shaurya Sharma, Taek Sang Yoon, Yizhak Kupfer

**Affiliations:** 1 Internal Medicine, Maimonides Medical Center, Brooklyn, USA; 2 Pulmonary and Critical Care, Maimonides Medical Center, Brooklyn, USA; 3 Critical Care, Maimonides Medical Center, Brooklyn, USA

**Keywords:** losartan, severe acute liver injury

## Abstract

Medications have been known to cause adverse drug reactions that affect various organs; these are mostly reversible reactions that improve with the cessation of the culprit medication. Losartan is an angiotensin-one receptor blocker which has been approved by the Food and Drug Administration (FDA) for the treatment of arterial hypertension. Fatigue, anemia, weakness, and cough are some of the common adverse effects of losartan. Acute hepatic injury has rarely been reported as an adverse effect of losartan. We report a case of a 61-year-old female with severe hepatic injury secondary to losartan use. None of the cases reported so far had such a high elevation of liver enzymes as seen in our patient.

## Introduction

Losartan is an angiotensin two type one (AT1) receptor antagonist which has been approved for the management of arterial hypertension. Losartan is also used in diabetics to prevent renal damage, and in patients with heart failure with reduced ejection fraction. Commonly reported side effects of losartan are dry cough, hyperkalemia, and muscle cramps. Losartan-induced acute liver injury has rarely been reported. We report a case of acute fulminant liver injury due to losartan, which has never been reported before to the best of our knowledge. 

## Case presentation

**Figure 1:**
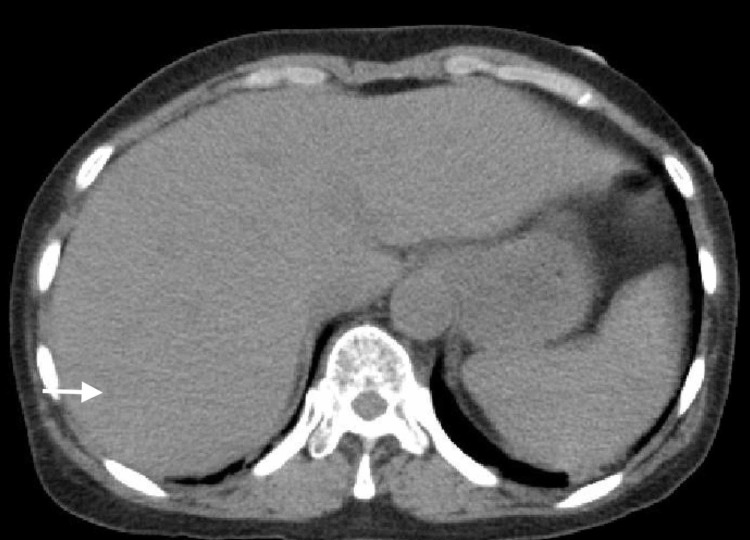
Computed tomographic scan of the abdomen showing mild hepatic steatosis

A 61-year-old woman with a history of hypertension, diabetes, and hypothyroidism presented to the emergency room with a high-grade fever, lethargy, and loss of appetite for the past couple of days. The patient had taken an over-the-counter Chinese cold preparation containing tylenol and diphenhydramine but without much relief. On the day of admission, she also vomited twice and was extremely dizzy. On initial presentation in the emergency room, the patient was febrile to 101.1 °F, respiratory rate was 18 breaths per minute, and blood pressure was 112/59 mm Hg. Blood work was significant for an abnormal liver profile with alanine transaminase (ALT) level of 1371 IU/L (normal 6-47 IU/L) and aspartate transaminase (AST) level of 1315 IU/L (normal 10-33 IU/L). Total and direct bilirubin was 0.7 mg/dl and 0.1 mg/dl respectively. Alkaline phosphatase (ALP) level of 75 IU/L was within normal limits. Computed tomographic (CT) scan of the abdomen and pelvis without oral or intravenous contrast revealed only mild hepatic steatosis as shown in Figure 1.

Due to elevated transaminitis, the patient was started on N-acetylcysteine (NAC) and liver function was closely monitored. All the home medications, including losartan and amlodipine, was held. On further blood work-up, serum amylase and lipase level were within the normal range, negative hepatitis panel (including A, B, and C), negative anti-mitochondrial and anti-smooth muscle antibodies and normal levels of serum alpha-one anti-trypsin and serum ceruloplasmin level. With ongoing treatment, the patient started improving clinically with down trending of AST and ALT level to 215 IU/L and 444 IU/L, respectively. The trend of the patient’s liver enzymes shown in Table 1.

**Table 1:** Course of patient’s liver enzymes ALT: Alanine transaminase; AST: Aspartate transaminase; ALP: Alkaline phosphatase

Liver enzymes	Day 1	Day 2	Day 3	Day 4
ALT (IU/L)	1,371	958	479	444
AST (IU/L)	1,315	831	308	215
ALP (IU/L)	75	65	80	101
Total Bilirubin (mg/dl)	0.7	0.4	0.3	0.3

The patient was discharged on day five, with down-trending of the liver enzymes. Losartan was among the differentials of the acute liver insult and the patient and the family were counseled to refrain from its further use.

The patient was brought back to the emergency room two days later with severe lethargy vomiting. Laboratory work-up showed ALT 3444 IU/L and AST 5232 IU/L. On further questioning, the patient’s family mentioned that they gave two doses of 100mg of losartan. NAC was immediately initiated and losartan was stopped on readmission. Over the course of a few days in the hospital, liver enzymes started trending down as shown in Table 2. 

**Table 2:** Course of patient’s liver enzymes on re-admission ALT: alanine transaminase; AST: aspartate transaminase; ALP: alkaline phosphatase

Liver enzymes	Day 1	Day 2	Day 3	Day 4
ALT (IU/L)	2,838	3,444	4,137	2,855
AST (IU/L)	3,600	5,232	5,677	2,905
ALP (IU/L)	161	62	161	133
Total bilirubin (mg/dl)	0.7	1.2	2.5	3.0

The patient and the family were counseled to strictly refrain from any more use of losartan. 

## Discussion

Losartan is a competitive antagonist and blocks angiotensin-one receptors. In 1995, the Food and Drug Administration (FDA) approved losartan for the treatment of arterial hypertension [1]. It was also approved for reducing risks of cerebrovascular accidents in patients with hypertension or left ventricular hypertrophy, and for slowing the progression of diabetic nephropathy by improving proteinuria [1]. Losartan has been studied extensively for the management of the aforementioned conditions. Losartan also has had a modest uricosuric effect and is used as a first-line agent for managing hypertension in patients with gout. 

The oral bioavailability of losartan is around 33% and it has a great first-pass metabolism [2]. Cytochrome P450 enzymes, CYP2C9 and CYP3A4, are involved in activating losartan to its active metabolite [2]. Losartan is mainly eliminated through feces and urine. The half-life of losartan is around two hours while the terminal half-life of its active metabolites is around six to nine hours. 

Drug-induced adverse reactions have been reported since the advent of the medication. Many drugs have been known to cause acute liver injury, and it has been labelled as one of the prime causes of liver failure in the country, requiring liver transplantation in over 75% of these patients [3]. The incidence of drug-induced liver injury (DILI) is around one to two cases per 1,00,000 person-years in the general population [4]. Abnormalities in the liver enzymes, especially alanine transaminase (ALT), aspartate transaminase (AST), alkaline phosphatase (ALP), gamma-glutamyl transpeptidase (GGT), and total and direct bilirubin are important tools for diagnosing a patient with DILI. Patients usually complain of malaise and jaundice upon initial presentation. Drug-induced liver injury can be hepatocellular, cholestatic, or mixed. Hepatocellular injury can result in a marked elevation of only the aminotransferases without significantly affecting the ALP or the total or direct bilirubin, while in cholestatic injury, the ALP is mainly affected and rises disproportionately to AST/ALT. In a mixed pattern, usually, a mixture of both the hepatocellular and cholestatic injury is observed. The 'R ratio', which is the ratio of serum ALT to serum ALP results with respect to their upper limits of normal (ULN), can also be used to label the type of liver injury [4]. An R ratio of five or more implies hepatocellular injury, two or lesser points towards cholestatic damage and values between two to five are labelled as mixed [4]. The use of the R ratio is often advised at the initial presentation. Liver biopsy is the gold standard for accurately classifying the type of injury. The results of the liver biopsy may vary with the timing of the biopsy as hepatocellular damage is more pronounced in the initial days/weeks while cholestatic damage becomes evident a little later [4].

To the best of our knowledge, 18 cases of angiotensin receptor blocker causing liver injury has been reported. Out of those, six cases were due to losartan, five were with Irbesartan, four with candesartan, and three cases were due to valsartan use as listed in Table 3 [1,5-10].

**Table 3:** All reported cases of liver injury due to angiotensin receptor blockers ALT: alanine transaminase; AST: aspartate transaminase

Case	Age/Sex	Drug/dose	Timing	AST (IU/L)	ALT (IU/L)	Re-exposure	Biopsy
1.	65 female	Losartan 50 mg	4 months	1,018	1,184	No	No
2.	46 male	Losartan 50 mg	1 month	2,042	2,547	No	No
3.	46 female	Losartan 50 mg	3 months	300	311	No	No
4.	55 female	Losartan 50 mg	3 weeks	635	650	No	No
5.	52 female	Losartan 50 mg	5 months	1,093	941	Yes	Yes
6.	77 male	Losartan 150 mg	3 weeks	115	410	No	Yes
7.	69 male	Irbesartan 300 mg	1 month	821	1,449	No	Yes
8.	59 NA	Irbesartan 300 mg	20 days	1,347	1,378	No	No
9.	51 female	Irbesartan 150 mg	3 weeks	237	890	No	No
10.	62 female	Irbesartan 300 mg	1 month	177	NA	No	Yes
11.	56 male	Irbesartan 300 mg	8 days	1,438	2,646	No	Yes
12.	41 female	Candesartan 16 mg	6 months	1,600	2,700	No	No
13.	61 female	Candesartan 16 mg	1 month	1,367	918	No	Yes
14.	82 male	Candesartan 16 mg	3 weeks	111	272	No	No
15.	70 female	Candesartan NA	2 weeks	441	244	No	No
16.	52 female	Valsartan NA	1 month	1292	780	No	No
17.	54 female	Valsartan 80 mg	5 months	738	1,664	No	Yes
18.	47 male	Valsartan 80 mg	2 weeks	360	776	No	No
OUR CASE	61 female	Losartan 100 mg	16 days	5,677	4,137	Yes	No

Although hepatocellular injury was the most common type of damage in the cases reported, cholestatic as well as mixed injury patterns were observed as well. 

We are reporting the seventh case of losartan-associated severe liver injury. Our patient also had hepatocellular injury as was evident by the elevated transaminases to around 30 times the upper limit of the normal range without any increase in serum ALP or bilirubin. Upon subsequent presentation 48 hours later, our patient was noted to be very lethargic. The family had mentioned that the patient took two doses of losartan 100 mg; the liver enzymes were found to be elevated to around 75-100 times the upper limit of the normal range with AST of 5,677 IU/L and ALT of 4,137 IU/L. This degree of elevation has never been reported before to the best of our knowledge. As per the Naranjo algorithm, the event scored a nine, which solidifies losartan as the 'definite cause' of acute liver injury. The usual cause of such high elevation in aminotransferases is shock liver. Other causes like viral hepatitis, tylenol toxicity, biliary blockage, and sepsis should also be ruled out. Disorders like Wilson’s disease, hemochromatosis, and autoimmune hepatitis can also lead to an elevation in aminotransferases.

Stopping the offending agent should be the first step as soon as the drug has been labelled as the cause of liver injury. N-acetyl cysteine can be used if the aminotransferase has been dangerously high or is not trending down. Usually, the improvement in the liver enzymes happen within hours to days but in a minority of patients, the liver damage progresses to necessitate liver transplantation. The degree of liver injury is usually worse when the patient is re-exposed to the same drug. The patient should be strictly counseled to not use the drug again. 

## Conclusions

Losartan and other angiotensin receptor blockers have very rarely been reported to cause acute liver injury. By reporting this case, we aim to increase awareness among physicians to always rule out losartan as a cause of unexplained elevation in aminotransferases. We also want to emphasize that once these drugs have been found to be the causative agent for the insult, patients should be strictly counselled not to use them again, as the acute liver injury may advance unto the need for liver transplantation. 
